# Treatment with penicillin G and hydrocortisone reduces ALS-associated symptoms: a case series of three patients

**DOI:** 10.12688/f1000research.10534.1

**Published:** 2017-04-03

**Authors:** Bert Tuk, Harmen Jousma, Pieter J. Gaillard

**Affiliations:** 1 Ry Pharma, Hofstraat 1, Willemstad, 4797 AC, Netherlands; 2 2-BBB Medicines, J.H. Oortweg 19, Leiden, 2333 CH, Netherlands

**Keywords:** Amyotrophic lateral sclerosis, dysphagia, dysarthria, penicillin G, hydrocortisone, GABA, neuromuscular disease, respiratory depression

## Abstract

Three male Caucasian patients with ALS were admitted to the hospital due to progressive dysphagia and dysarthria.

During two 21-day courses of penicillin G and hydrocortisone, these patients’ dysphagia and dysarthria resolved. The patient’s other ALS-associated symptoms also improved, including respiratory function, coordination, walking, and muscle strength.

This is the first report of a treatment with a protocol for treating dysphagia, dysarthria, respiratory depression and other ALS-related symptoms. Furthermore, the observations are consistent with the recent hypothesis that the successful treatment of ALS symptoms with this treatment course in six patients with syphilitic ALS was not directly due to the treatment of syphilis; but that the administered penicillin G and/or hydrocortisone treated these patients’ ALS symptoms due the off-target pharmacological activity of penicillin G and/or hydrocortisone. This report therefore underscores the need to evaluate the efficacy of this treatment course in a clinical trial.

## Introduction

Amyotrophic lateral sclerosis (ALS, also known as Lou Gehrig’s disease) is a rapidly progressive devastating disease with an average life expectancy of only 3–5 years after diagnosis ^1– 3^. The cumulative lifetime risk of ALS is approximately 1:350–400 ^3^. Moreover, the total cost associated with ALS—excluding the cost of medication—has been estimated to exceed $1.4 million per patient ^4^. The clinical manifestations of ALS include progressive wasting of muscle mass, reduced muscle coordination, dysphagia, dysarthria, and fatal respiratory depression ^1– 3^. Several observations regarding the putative pathogenesis of ALS have been reported ^1– 3^; however, although more than 140 years have passed since ALS was first described, its pathogenesis remains poorly understood, and no disease-modifying treatment is available.

Dysphagia-associated aspiration pneumonia is the leading cause of death in many neuromuscular and neurological diseases, including ALS, Parkinson’s disease, and Alzheimer’s disease ^5, 6^. Remarkably, however, no effective treatment for dysphagia is currently available.

Here, we report that dysphagia, dysarthria, and other ALS-related symptoms resolved during two 21-day courses of penicillin G and hydrocortisone. This treatment course was previously reported to be efficacious in six patients with so-called syphilitic ALS in which syphilis was hypothesized to cause ALS ^7, 8^. However, we recently proposed that the treatment effect was not due to the treatment of syphilis, but rather was a consequence of the multifaceted pharmacology of penicillin G and/or hydrocortisone ^9^. Given that our three patients presented with no evidence of syphilis, our results provide evidence that treating ALS patients with a course of penicillin G and hydrocortisone—regardless of whether they present with syphilitic ALS or non-syphilitic ALS—may effectively treat the symptoms of this rapidly progressive disease. These three cases warrant further study of this treatment course.

## Case presentation: Patient 1

A 42-year-old Caucasian male with ALS was admitted to the hospital with complaints of progressive swallowing and speech difficulties. Three years ago, this patient was diagnosed with limb-onset ALS and presented with other symptoms typical of ALS, including dysphagia, dysarthria, difficulty with coordination, and muscle wasting. The patient had no history of syphilis or other systemic infection, and the diagnosis of ALS was confirmed by the Netherlands National ALS Center.

**Figure 1.**
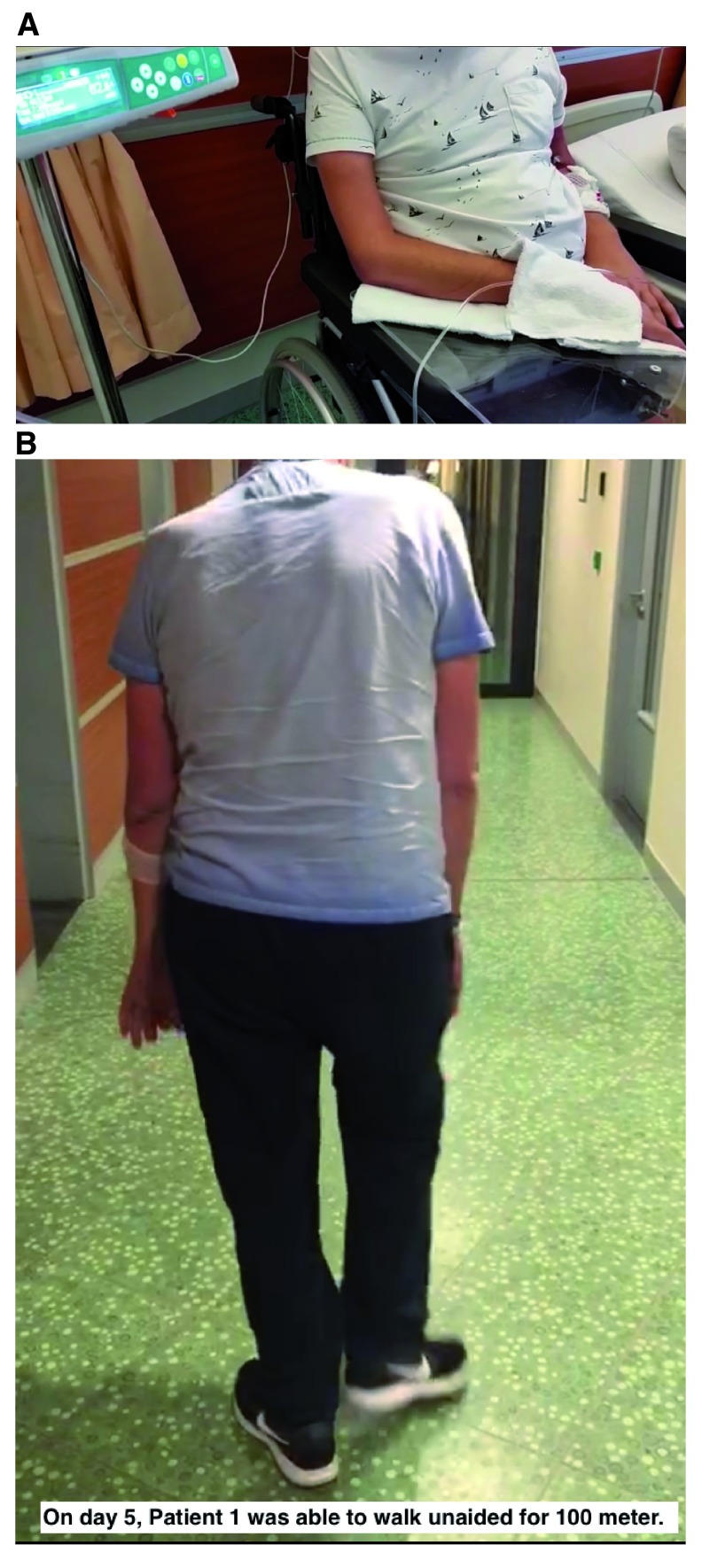
**A:** Upon admission to the hospital, patient 1 was only able to take a few steps and had been wheelchair-bound for the past four months. In addition, his upper extremities had been paralyzed for over twelve months. In the preceding months, the patient’s speech had degenerated, and the patient had difficulty swallowing both solid food and liquids, including saliva. **B:** On the 2 ^nd^ and 3 ^rd^ treatment days, patient 1 reported that he was able to lie in bed without experiencing muscle pain in the neck, shoulders, or back. On day 4, the patient was able to stand from a sitting position. On day 5, the patient was able to walk unaided a distance of approximately 100 meters. During days 76 to 91 the patient experienced increasing muscle pain in the neck, shoulders, or back, his walking ability regressed, and the patient became wheelchair-bound again. His swallowing and speech remained functional.

Upon admission to the hospital, the patient was only able to take a few steps and had been wheelchair-bound for the past four months. In addition, his upper extremities had been paralyzed for over twelve months (see Figure 1A). In the preceding months, the patient’s speech had degenerated, and the patient had difficulty swallowing both solid food and liquids, including saliva.

**Figure 2.**
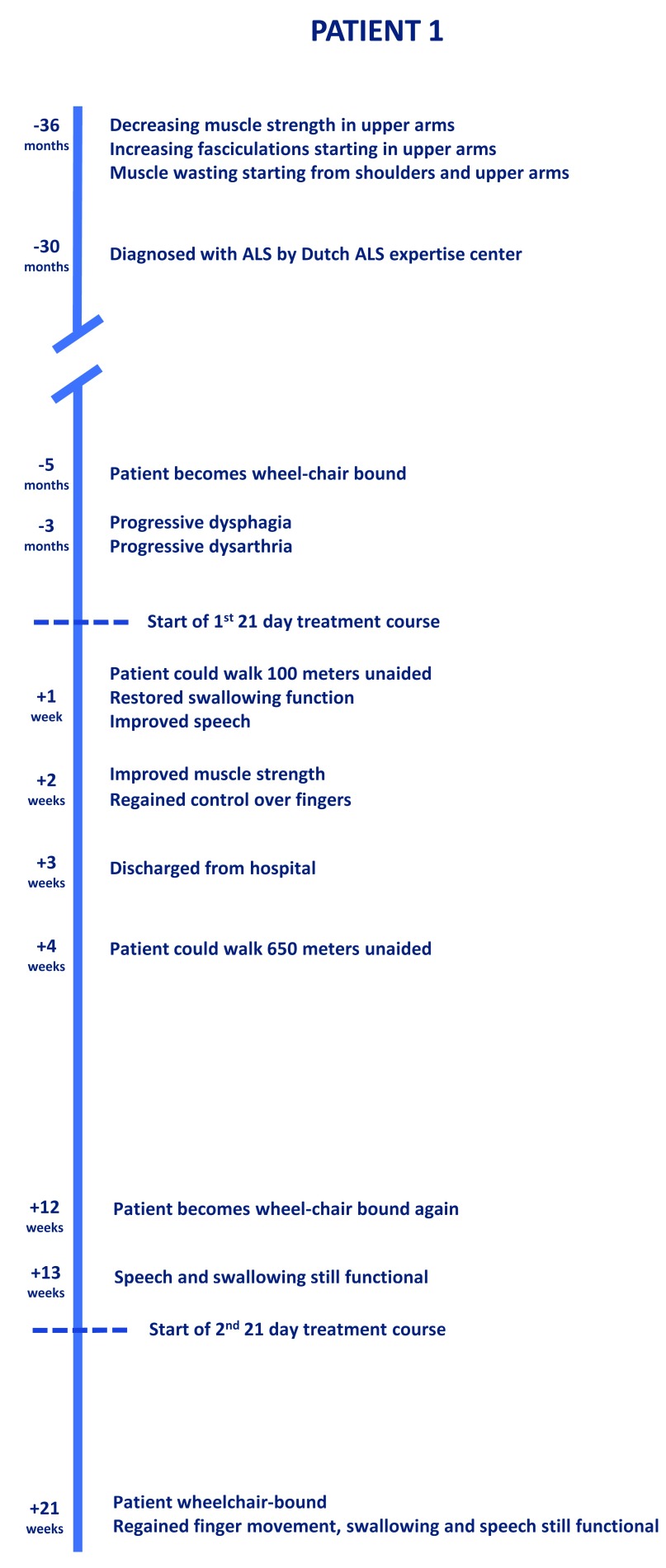
The clinical progression and effect of treatment in Patient 1.

Figure 2 shows the progression of symptoms and the effect of treatment. Dysphagia was confirmed at the time of admission by performing a fiber-optic endoscopic evaluation of swallowing (FEES) examination ^10^ ( Movie 1). Physical examination and laboratory blood analysis revealed no other clinical pathology, and renal function was normal. The patient was not taking any prescription medications.

The patient had no history of seizures and was therefore eligible to receive high doses of penicillin G. After confirming that the patient was not allergic to penicillin (by administering a daily dose of amoxicillin for six days), the patient was started on a 21-day course of penicillin G and hydrocortisone ( Table 1) delivered via midline catheter infusion. This treatment course was recently postulated to be efficacious for treating dysphagia, dysarthria, and other ALS-related symptoms ^9^.

**Table 1.** 21-day course of penicillin G and hydrocortisone consisting of an 8-hour infusion on each treatment day.

*Treatment* *day(s)*	*8-hour continuous infusion*
1	1 million units penicillin G + 100 mg hydrocortisone
2	3 million units penicillin G + 100 mg hydrocortisone
3	5 million units penicillin G + 100 mg hydrocortisone
4	10 million units penicillin G + 100 mg hydrocortisone
5 – 14	20 million units penicillin G + 100 mg hydrocortisone
15 – 21	20 million units penicillin G

On the 2 ^nd^ and 3 ^rd^ treatment days, the patient reported that he was able to lie in bed without experiencing muscle pain in the neck, shoulders, or back. On day 4, the patient was able to stand from a sitting position. On day 5, the patient was able to walk unaided a distance of approximately 100 meters (see Figure 1B). In addition, the patient’s speech and swallowing improved.

At the end of the first week, the patient’s dysphagia and dysarthria symptoms had resolved fully, and his walking had improved further. On day 9, the patient was able to move the fourth and fifth fingers on his right hand for the first time in a year, and by day 11 he had regained control of these two fingers ( Movie 2). On day 11, a repeat FEES examination confirmed that the patient’s dysphagia had resolved. On day 12, the patient was able to move all of the fingers on his right hand ( Movie 3) and grasp objects with his left hand, and he could once again operate the mouse attached to his computer. Furthermore, the patient’s voice recognition software was able to interpret the speech of the patient for the first time in months. On day 14, it was possible to sample venous blood from the patient’s forearm for the first time since he was admitted to the hospital. On day 18, the patient’s motor function further improved ( Movie 4).

At the end of day 21, physiological and FEES examinations revealed that swallowing function remained intact ( Movie 5), and the patient reported that his breathing and sleep quality had improved markedly during the treatment course. Therefore, in accordance with the defined treatment protocol, the midline catheter was removed and the patient was discharged. Upon discharge, the patient’s speech was restored to nearly pre-ALS levels, and he had regained the ability to stand from his wheelchair and walk unaided. The patient had also regained control over his fingers and had regained the ability to grasp light objects. Overall muscle function and strength were also improved as evidenced by increased power in his arms and legs and his renewed ability to stand, walk, and bend at the waist. Furthermore, his respiratory function had improved. Lastly, the patient reported that his general muscle pain had regressed, and the pain in his shoulder muscles had resolved completely.

After returning home, the patient continued to improve. On day 22 (the first day following the end of the treatment course), the patient was able to lie in a dentist’s chair for 40 minutes without muscle pain, which had not been possible prior to receiving the treatment, and the patient was able to complete the dental procedure. On day 25, the patient was able to walk unaided a distance of approximately 650 meters. During the follow-up period from day 25 through day 75, the patient had generally stabilized. During days 76 to 91 the patient experienced increasing muscle pain in the neck, shoulders, or back, he no longer could operate the mouse attached to his computer, his voice recognition software no longer was able to interpret his speech, his walking ability regressed to the point that he not even take a few steps, and the patient became wheelchair-bound again. His swallowing and speech remained functional.

On day 92, the patient was readmitted to the hospital and started on a second 21-day course of penicillin G and hydrocortisone ( Table 1) delivered via midline catheter infusion. Physiological and FEES examination on day 92 revealed that swallowing function had remained stable relative to day 21. During the 2 ^nd^ 21-day course (days 92 to 113), the patient reported that he was able to lie in bed without experiencing muscle pain in the neck, shoulders, or back, and that his walking ability had slightly improved. Furthermore, his speech and swallowing function had improved. During days 114 to 150 the patient remained wheelchair-bound. His regained finger movement, swallowing and speech remained functional.

## Case presentation: Patient 2

A 51-year-old Caucasian male with ALS was admitted to the hospital with complaints of progressive swallowing and speech difficulties. One year earlier, this patient was diagnosed with bulbar-onset ALS and presented with symptoms typical of bulbar-onset ALS, including dysphagia and dysarthria. Over the previous year, the patient also developed other symptoms typical of ALS, including fasciculations, coordination problems, and muscle weakness. Furthermore, the patient experienced tremor-like movements when lying in bed and when getting up in the morning. These transient muscle tremors prevented the patient from standing and walking directly after getting out of bed. The patient also experienced bladder control problems for the previous two years, but tested negative for bladder infection. The patient had no history of syphilis or other systemic infection, and the diagnosis of ALS was confirmed by the Netherlands National ALS Center.

**Figure 3.**
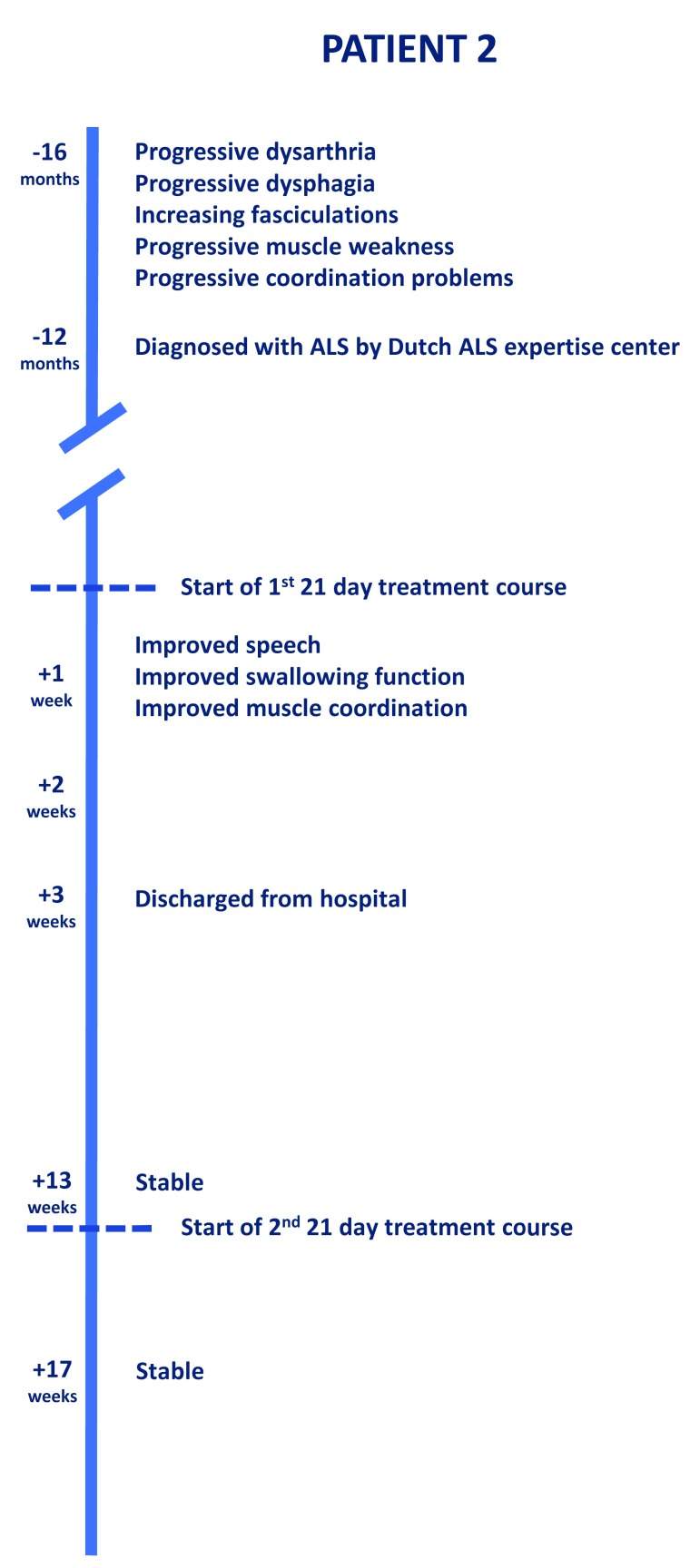
The clinical progression and effect of treatment in Patient 2.

In the year preceding admission to the hospital, the patient’s speech had degenerated, and the patient had difficulty swallowing both solid food and liquids, including saliva, leading to the manifestation of a high frequency of coughing. Figure 3 shows the progression of symptoms and the effect of treatment in Patient 2. Dysphagia was confirmed by FEES examination at the time of admission ( Movie 6), and speech impairment was confirmed by a speech therapist. Physical examination and laboratory blood analysis revealed no other clinical pathology and normal renal function; a test for syphilis was negative. The only prescription medication taken by the patient was Riluzole (100 mg/day).

The patient had no history of seizures and was therefore eligible to receive high doses of penicillin G. After confirming that the patient was not allergic to penicillin (as described for Patient 1), the patient was started on a 21-day course of penicillin G and hydrocortisone ( Table 1) delivered via midline catheter infusion.

On the 4 ^th^ treatment day, the frequency and severity of coughing had decreased markedly, and a slight improvement in speech was noted. Furthermore, bladder function had improved. On day 5, the patient’s speech further improved, and the patient had stopped coughing completely, indicating improved swallowing function. From day 6 onwards, swallowing and speech function were further improved, as confirmed by FEES examination ( Movie 7) and evaluation by a speech therapist, both on day 20. Furthermore, respiratory function improved. On days 11–12, the patient’s blood pressure had increased, and the patient reported a severe headache, both of which resolved after starting treatment with a blood pressure medication (nifedipine). After the discontinuation of hydrocortisone on day 15, the patient no longer reported headache symptoms, and his blood pressure had normalized. On days 16 and 17, the patient reported slight muscle weakness in his legs, which resolved within a few days. At the end of day 21, the midline catheter was removed and the patient was discharged. During the follow-up period from day 21 through day 91, the patient had generally stabilized. On day 92, the patient was readmitted to the hospital. Physiological and FEES examination on day 92 revealed that swallowing function had remained stable relative to day 21. The patient was started on a second 21-day course of penicillin G and hydrocortisone ( Table 1) delivered via midline catheter infusion. Because the observed blood pressure increase during the first course, the patient was administered 30 mg nifedipine retard once daily, starting from the first day of the 2 ^nd^ treatment course. On the 2 ^nd^ day of the 2 ^nd^ 21-day course (day 93), the patient’s blood pressure had increased, which resolved after increasing the nifedipine dose to 90 mg daily. During the second 21-day course, and during the follow-up period from day 112 to 122, the patient remained stable.

## Case presentation: Patient 3

A 65-year-old Caucasian male with ALS was admitted to the hospital with severely impaired swallowing and speech. One year earlier, this patient was diagnosed with bulbar-onset ALS and presented with symptoms typical of bulbar-onset ALS, including dysphagia and dysarthria. Over the course of the disease, the dysphagia had progressed to the level that the patient’s oral intake had become inadequate, and the patient was scheduled to undergo a percutaneous endoscopic gastrostomy (PEG). In the year prior to admission to the hospital, the patient’s speech also had become severely impaired, and the patient developed other symptoms typical of ALS, including coordination problems, muscle weakness, cramps and leg resting tremors. A few months before admission, the patient could no longer walk unaided and began using a walker. The patient had no history of syphilis or other systemic infection, and the diagnosis of ALS was confirmed by the Netherlands National ALS Center.

**Figure 4.**
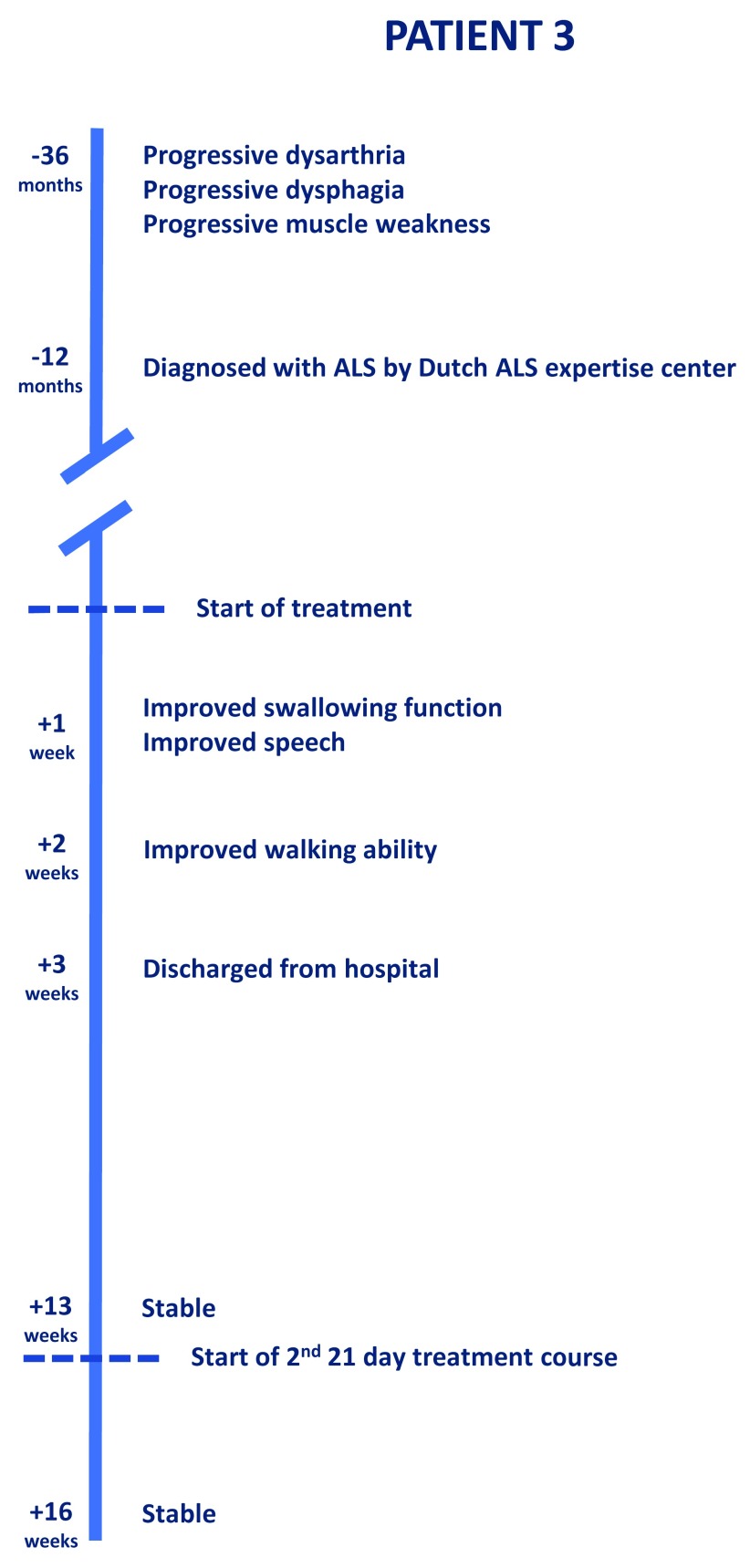
The clinical progression and effect of treatment in Patient 3.

Figure 4 shows this patient’s progression of symptoms and the effect of treatment. Dysphagia was confirmed by FEES examination at the time of admission ( Movie 8), and the speech impairment was confirmed by a speech therapist. Physical examination and laboratory blood analysis revealed no other clinical pathology and normal renal function. The only prescription medication taken by the patient was losartan (100 mg/day) for high blood pressure.

The patient had no history of seizures and was therefore eligible to receive high doses of penicillin G. After confirming that the patient was not allergic to penicillin (as described for Patient 1), the patient was started on a 21-day course of penicillin G and hydrocortisone ( Table 1) delivered via midline catheter infusion.

On the 3 ^rd^ treatment day, the patient was able to drink a glass of water for the first time in months. On day 4, swallowing function improved further, and the patient was able to swallow solid food for the first time in months. Furthermore, the patient’s speech improved, his muscle stiffness has diminished, and he no longer experienced cramps or leg rest tremors. Over the following days, both swallowing and speech function continued to improve, as confirmed by FEES examination ( Movie 9) and evaluation by a speech therapist. From day 7 onwards, the patient’s swallowing function continued to improve to the point that a PEG procedure was no longer necessary, and his speech further improved. On day 8, the patient reported increased muscle strength in arms and legs, and the patient’s walking ability had improved. Furthermore, his weight had increased with 3 kg, and his respiratory function improved. On day 9, the patient’s blood pressure had increased, and the patient reported a headache. He was therefore placed on blood pressure medication (nifedipine), and the hydrocortisone was discontinued on day 10 (rather than on day 14 as indicated in the protocol). After hydrocortisone was discontinued, the patient no longer reported headache symptoms, and blood pressure was normalized. On days 11 and 12, the patient reported slight muscle weakness in his legs, which resolved within a few days. At the end of day 21, the midline catheter was removed and the patient was discharged. During the follow-up period from day 21 through day 90, the patient had generally stabilized. On day 91, the patient was readmitted to the hospital and was started on a second 21-day course of penicillin G and hydrocortisone ( Table 1) delivered via midline catheter infusion. Because the observed blood pressure increase during the first course, the patient was administered 30 mg nifedipine retard once daily. During the second 21-day course (days 91 to 112) the patient remained stable. 

## Discussion

Here, we report that two 21-day courses of penicillin G and hydrocortisone treated symptoms typical of ALS in three patients with no history of syphilis. After only four days of treatment, Patient 1—who had been wheelchair-bound for four months—was able to walk unaided approximately 100 meters. In addition, this patient rapidly regained movement of his fingers and could grasp items with his left hand, functions that had been completely absent for eight months prior to treatment. Furthermore, the symptoms in Patient 2—who had experienced progressive dysarthria, dysphagia, and bladder dysfunction over the preceding year—also regressed during the first week of treatment. Lastly, in Patient 3—who presented with dysphagia so severe that PEG was indicated—both the dysphagia and dysarthria regressed during the first week of treatment.

The treatment protocol was generally well tolerated by all three patients. The only side effect observed in Patient 1 was slight, transient edema of the arm and hand at the site where the midline catheter was placed. Patient 2 experienced high blood pressure and a severe headache beginning on day 11; these symptoms can be attributed to the administration of hydrocortisone, and they resolved after the discontinuation of hydrocortisone. Similarly, patient 3 experienced high blood pressure on day 9, which resolved after the discontinuation of hydrocortisone on day 10. During the second course of treatment, the increase in blood pressure in these patients was effectively treated with nifedipine. These observations indicate that blood pressure should be monitored closely during the treatment protocol. Patients 2 and 3 experienced temporary muscle weakness in their legs in the days following the discontinuation of hydrocortisone. Because Patients 2 and 3 are not in the late stages of ALS disease, in which respiratory muscle function is often severely affected, the hydrocortisone withdrawal–induced temporary muscle weakness was well tolerated. However, in late-stage ALS, in which muscle function is often reduced to minimal functional levels, hydrocortisone withdrawal–induced muscle weakness may lead to serious side effects, including respiratory depression.

The absence of seizure activity in these patients—even on high-dose penicillin G—is consistent with our recent observation that seizures are often absent in patients with ALS ^11^. In the treatment of syphilis, for which this treatment protocol was originally developed, increasing doses of penicillin G are given in order to minimize the risk of inducing a Jarisch-Herxheimer reaction. However, this titration of penicillin G is also recommended for use in ALS patients, as these patients may have impaired blood-brain barrier (BBB) function ^12, 13^, potentially resulting in high levels of penicillin G in the CSF, which could induce seizure activity. In this respect, including hydrocortisone in the treatment course may provide additional benefits, as hydrocortisone has been reported to maintain BBB integrity ^14^.

Four courses of the 21-day treatment protocol reported here was previously reported to be efficacious in treating six cases of so-called syphilitic ALS ^7, 8^. Syphilitic ALS is an intriguing clinical phenomenon, as it is the only form of ALS ever reported to have been cured ^9^. Recently, we proposed that the successful treatment of ALS symptoms in these six patients with syphilitic ALS was not directly due to the treatment of syphilis; specifically, we proposed that the penicillin G and/or hydrocortisone treated these patients’ ALS symptoms due to the off-target pharmacological activity of penicillin G (e.g., as a GABA receptor antagonist) and/or the multifaceted pharmacological activity of hydrocortisone (e.g., as an immunosuppressant) ^9^. This notion is supported by the three cases reported here, as our patients had no syphilis-related symptoms or history of syphilis.

It is important to note that either penicillin G or hydrocortisone—or both—contributed to the observed effects. Penicillin G is a GABA receptor antagonist ^15^ that can reduce GABAergic overstimulation. At high doses, penicillin G can also affect other major bodily functions and/or systems, including the immune system, the cardiovascular system, metabolic function, renal function, liver function, the hematological system, and the urogenital system (penicillin G summary of product characteristics, see https://www.medicines.org.uk/emc/medicine/2962. Accessed July 27, 2016); these pharmacological activities may be associated with the clinical benefits reported here.

Hydrocortisone has immunomodulatory and anti-inflammatory properties (hydrocortisone summary of product characteristics. https://www.medicines.org.uk/emc/medicine/10815, accessed July 27, 2016, and hydrocortisone sodium succinate product monograph. https://www.drugs.com/monograph/hydrocortisone-sodium-succinate.html, accessed November 16, 2016); therefore, it may also affect systems involved in the pathogenesis of ALS, including the anti-inflammatory system ^1– 3^. Moreover, hydrocortisone has reported efficacy in treating multiple sclerosis and respiratory diseases (hydrocortisone summary of product characteristics. https://www.medicines.org.uk/emc/medicine/10815, accessed July 27, 2016), conditions that have clinical overlap with ALS. Furthermore, like penicillin G, hydrocortisone can affect several bodily functions and/or systems, including the endocrine system, the immune system, inflammatory function, the respiratory system, the hematological system, and the gastrointestinal system (hydrocortisone summary of product characteristics. https://www.medicines.org.uk/emc/medicine/10815, accessed July 27, 2016). Furthermore, glucocorticoids have been found to be efficacious in preclinical models of ALS ^16^.

Other explanations may account for the observations reported here. First, the patients may have improved due to a placebo effect. However, this may be deemed unlikely, as the full range of ALS symptoms resolved in these patients. Nevertheless, the possibility that the patients improved due to a placebo effect should be investigated in a clinical trial setting. Second, the patients may have been incorrectly diagnosed as having ALS and/or incorrectly diagnosed as not having neurosyphilis. However, this is also unlikely, given that Patient 2 tested negative for syphilis before starting treatment, and given that all three patients were diagnosed at a leading neurology center with extensive experience diagnosing both ALS and neurosyphilis. Furthermore, syphilis has an extremely low prevalence in the Netherlands (present in only 0.15% of the population) ^17^, and the symptoms associated with ALS generally do not overlap with the symptoms associated with syphilis ^18^. Third, it is possible that the patients’ ALS symptoms were caused by an infection other than syphilis, which was then treated by the penicillin and hydrocortisone. The possible presence of an unidentified infection—and its treatment with penicillin G—may explain the observation that the benefits of treatment remained even after treatment was discontinued. This is an interesting point, as the elimination half-life of penicillin G and hydrocortisone is 0.5–1.0 hour (penicillin G summary of product characteristics, see https://www.medicines.org.uk/emc/medicine/2962, accessed July 27, 2016) and 1.5–3.5 hours (hydrocortisone sodium succinate product monograph. https://www.drugs.com/monograph/hydrocortisone-sodium-succinate.html, accessed November 16), 2016, respectively; thus, both compounds would have been cleared from the body within hours after treatment was discontinued. Nevertheless, given the apparent absence of infection in all three patients, we believe that the post-treatment effects were due to the treatment’s effects on ALS rather than its antibacterial activity. Finally, it is formally possible that Patient 2 may have improved because of the concomitant administration of Riluzole. However, this is unlikely, as this patient’s symptoms had been progressing steadily since he was first diagnosed with ALS, and he was on Riluzole since he was diagnosed. Moreover, this patient began to improve only after he started on the penicillin and hydrocortisone protocol.

## Conclusions

This is the first report of a treatment with a protocol for treating dysphagia, dysarthria, and other ALS-related symptoms using a 21-day course of penicillin G and hydrocortisone in three patients with ALS and no history or symptoms of syphilis. This is an important clinical observation, as no treatment is currently available for dysphagia, dysarthria, or ALS. Furthermore, the findings support the recent hypothesis that the successful treatment of ALS symptoms with this treatment course in six patients with syphilitic ALS was not directly due to the treatment of syphilis; but that the administered penicillin G and/or hydrocortisone treated these patients’ ALS symptoms due the off-target pharmacological activity of penicillin G and/or hydrocortisone. In view of the devastating, rapidly progressive nature of ALS, this treatment protocol should be evaluated further in a clinical trial.

## Consent

Written informed consent for publication was obtained from the patients. The requirement for ethical approval was waived by the Medical Ethics Review Committee of the Academic Medical Center. Patients were treated according to guidelines of the Royal Dutch Medical Association (KNMG) for off-label prescribing (see https://protect-eu.mimecast.com/s/DdxhBlJkYHQ). Medication was administered under supervision of E.W.J. Wielinga, M.D., Ph.D., consultant ENT-surgeon, who also validated the results regarding swallowing and speech.

## Supplementary material

Movie 1: FEES in Patient 1 prior to the start of the treatment, showing impaired swallowing function while attempting to swallow two pieces of bread.

.

Movie 2: On day 11, Patient 1 had regained movement of the fourth and fifth fingers on his right hand.

.

Movie 3: On day 12, Patient 1 had regained movement of all four fingers on his right hand.

.

Movie 4: On day 18, Patient 1 could grasp objects.

.

Movie 5: FEES in Patient 1 on day 21, showing restored swallowing function. Note that the patient fully swallowed the piece of bread, and the patient could swallow faster than on day 0 (compare with Movie 1).

.

Movie 6: FEES in Patient 2 prior to the start of the treatment, showing impaired swallowing function while attempting to swallow two pieces of bread. Note that residue of bread is still present in the vallecula after 4 attempts of swallowing.

.

Movie 7: FEES in Patient 2 on day 20, showing improved swallowing function. After swallowing a piece of bread in one time, no residue can be discerned in the vallecula (compare with Movie 6).

.

Movie 8: FEES in Patient 3 prior to the start of the treatment showing that drinking water was impossible without chocking and coughing.

.

Movie 9: FEES in Patient 3 on day 16, showing functional swallowing of a piece of bread (compare with Movie 8).
