# Supplementary material for: Treatment with penicillin G and hydrocortisone reduces ALS-associated symptoms: a case series of three patients
Source: F1000Res. 2017 Apr 3;6:410. [Version 1] doi: 10.12688/f1000research.10534.1 (PMC5383939; doi:10.12688/f1000research.10534.1)
Supplement: Peer review history

# Peer review: Referee response for version 1

**DOI:** 10.5256/f1000research.11352.r21581

**Published:** 2017-04-05

**Authors:** Alan Gill, Fernando G. Vieira

**Affiliations:** 1 ALS Therapy Development Institute, Cambridge, MA, USA

Interpreting any therapeutic benefit of Penicillin G plus hydrocortisone on ALS symptom severity and progression does require a careful examination of drug effects that occur outside of the intended general GABA receptor antagonism and anti-inflammatory actions, as well as the effects of additional drugs the patients take, including those needed to offset untoward effects of the primary treatment, e.g., nifedipine. Plasma volume expansion by hydrocortisone might be expected to make venous blood sampling easier, but also to increase blood pressure to levels that need attention. While direct vasodilation by nifedipine increased the size of the circulation’s container enough to permit blood pressure to normalize, both nifedipine and hydrocortisone soon promote additional plasma volume expansion to fill the larger container. The authors do carefully document the application of all drugs in a way that permits therapists to anticipate managing these side-effects.

 The authors provide solid safety recommendations about dosing with both the Penicillin G and with the hydrocortisone. These recommendations would be important even without the potential for blood brain barrier leakiness and for adrenal dysregulation that can occur in ALS in order to prevent seizures and unexpected responses by the pituitary-adrenal axis. The authors formally recognize and take these potential risks into account.

 We do believe that the apparent therapeutic benefit of treatment shown by patients in the current report is interesting at face value. However, if the objective of the report is to convince ALS clinicians and patients to consider trying this off-label therapeutic approach, then the manuscript would be better served to include typical ALS endpoint data, e.g., ALS FRS scores, forced vital capacity (or slow vital capacity) measurements, and patient body weight, alongside the clinical description and videos. While ALS FRS is subjective, it is familiar to neurologists and patients and could provide important context for the magnitude of benefit observed. FVC/SVC and body weights are more objective measures that would provide indications about the patients’ well-being, although body weights would be influenced in a potentially confounding way by the hydrocortisone during, but perhaps not after, treatment. All of these endpoints are standard in ALS clinics, both in the United States and in Europe.

 One can hope that careful clinical testing of the current regimen might encourage efforts to try to identify an even safer, more predictable, therapeutic regimen than PENG/hydrocortisone itself. Such efforts might try to understand which inhibitory interneuron networks require interventions and which do not, and which specific antagonist (or agonist) drugs would optimize our test of the hypothesis. Can specific peptide neuromodulators contribute to improving the regimen? These considerations do not preclude the importance of carefully testing the PENG/hydrocortisone regimen itself. The complexity of this task alone is significant. Refinements of the regimen only add complexity, but could lead to more predictable, safer, and even more effective ALS treatment.

We have read this submission. We believe that we have an appropriate level of expertise to confirm that it is of an acceptable scientific standard.

# Peer review: Referee response for version 1

**DOI:** 10.5256/f1000research.11352.r21563

**Published:** 2017-04-05

**Authors:** Rien Vermeulen

**Affiliations:** 1 Academic Medical Center, Amsterdam, Netherlands

This is a remarkable report on three patients with ALS who improved after treatment with penicillin G and hydrocortisone. This treatment was based on the observed beneficial effects of this treatment in patients with ALS and syphilis. The authors hypothesize, this effect of treatment is a result of reduction of GABAergic overstimulation by penicillin G.

 However, all too often neurologists have seen astonishing improvements in uncontrolled studies. Therefore this treatment should be tested in a randomized controlled trial. Before embarking on a large scale trial, I would like to suggest to start with a small efficacy study in patients with ALS in whom the respiratory function is decreasing. The primary outcome in this trial could be the maximal inspiratory pressure which is a well known surrogate marker for disease progression in patients with ALS. In trials in patients with ALS few placebo-effects have been observed, which means that a quick answer is possible to the question whether or not this treatment improves respiratory function. Moreover, there are no safety issue in such a study.

 Clinicians owe to their patients with this devastating disease to test the effects of this treatment with high priority, since there are presently no other promising therapies.

I have read this submission. I believe that I have an appropriate level of expertise to confirm that it is of an acceptable scientific standard.
